# Case Report: The window that closed too soon: lessons from a late CLN2 diagnosis and death of a 9-year-old boy

**DOI:** 10.3389/fgene.2025.1622185

**Published:** 2025-07-04

**Authors:** Anna Bryzik, Dawid Larysz, Patrycja Larysz, Julia Izabela Karpierz, Justyna Paprocka

**Affiliations:** ^1^ Department of Pediatrics, Haematology and Pediatric Nephrology, Provincial Specialist Hospital, Częstochowa, Poland; ^2^ Department of Head and Neck Surgery for Children and Adolescents, Regional Specialized Children’s Hospital Popowski, University of Warmia and Mazury, Olsztyn, Poland; ^3^ Students’ Scientific Society, Pediatric Neurology Department, Faculty of Medical Sciences, Medical University of Silesia, Katowice, Poland; ^4^ Pediatric Neurology Department, Faculty of Medical Sciences, Medical University of Silesia, Katowice, Poland

**Keywords:** neuronal ceroidlipofuscinosis, batten disease, neurodegenerative disease, epilepsy, rare disease

## Abstract

A class of progressive, autosomal recessive neurodegenerative diseases known as neuronal ceroid lipofuscinoses (NCLs) are brought on by lysosomal protein or enzyme dysfunction. This leads to the pathological buildup of autofluorescent ceroid-lipofuscin in neurons and other tissues. In children and young adults, NCLs are the most frequent cause of dementia and neurodegeneration, and epilepsy, psychomotor regression, visual decline, ataxia, and early death are all examples of clinical characteristics which may appear in the disease’s natural course. Cerliponase alfa (known as Brineura) is an enzyme replacement therapy and has become increasingly important in treating CLN2 disease (late-infantile NCL), which is caused by a deficiency of the lysosomal enzyme tripeptidyl peptidase 1 (TPP1). When started early, it can significantly slow the progression of the disease. We describe the course of a boy’s diagnosis and treatment of CLN2 disease, which demonstrates the negative effects of delayed recognition. NCL was not suspected until more than a year after treatment-resistant epilepsy, progressive ataxia, and psychomotor decline appeared, despite early-onset speech delay, developmental variability, and epileptic seizures beginning at age 3. Clear abnormalities were not found by initial imaging or genetic testing. The child’s neurological decline was already severe by the time biochemical and molecular confirmation of CLN2 disease was obtained at the age of 5, and with increasing seizures, loss of motor and cognitive abilities, vision loss, gastrostomy, tracheostomy, and death at 9 years, he progressed according to the natural course of CLN2. Even with repeated medical contact and neurological evaluations, this case highlights the difficulty of diagnosing NCLs and the significant chance of missing the limited therapeutic window. It emphasizes how neurologists and pediatricians need to be more aware of NCLs as possible causes of developmental regression and early-onset epilepsy. Children with such presentations may benefit from earlier metabolic or enzyme testing, which could increase access to treatments that prolong life and change the disease’s deadly course.

## 1 Introduction

Neuronal ceroidolipofuscinoses (NCL), also known as Batten disease, is a heterogeneous group of congenital neurodegenerative disorders characterized by excessive accumulation of lipofuscin in various cells and organs, resulting from defective lysosomal processing enzymes or proteins (Rosenberg et al., 2019; Schulz et al., 2013). All types of NCL follow a progressive neurodegenerative course and represent the most common cause of dementia in children and young adults. The hallmark of the disease is neuronal involvement, with intracellular accumulation of lipofuscin-like storage material and ceroid leading to cellular damage. The primary clinical manifestations stem from progressive damage to the nervous system and retina, although the characteristic intracellular deposits can accumulate in cells throughout the body (Orlin et al., 2013; Kamate et al., 2012; Worgall et al., 2007). Most forms are inherited in an autosomal recessive (AR) manner, with the exception of a rare adult-onset variant (Parry-type NCL), and are associated with mutations in NCL-related genes (Bennett and Rakheja, 2013). The most commonly implicated genes include CLN1/PPT1, CLN2/TPP1, CLN3, CLN5, CLN6, CLN7/MFSD8, CLN8, and CLN10/CTSD. These genes encode enzymes or lysosomal proteins essential for maintaining proteostasis and autophagic function. Deficiency in these proteins causes progressive accumulation of ceroid-lipofuscin materials—particularly subunit c of mitochondrial ATP synthase—which leads to neurodegeneration. As of 2025, genotype–phenotype correlations have emerged from databases cataloguing over 500 known pathogenic variants, which result in varying degree of the disease severity, as well as age of onset and symptoms (Kousi et al., 2012; Kaminiów et al., 2022; Cotman et al., 2015; Mole, 2004; Cotman et al., 2013). NCL pathogenic variants include missense, nonsense, splicing mutations, small insertions/deletions, and larger rearrangements. The mutation-specific effect on protein includes complete loss of function, partial deficiency, and varying secretion or localization abnormalities (Mole, 2004; Cotman et al., 2013; Gardner and Mole, 2021; Palmer et al., 2013; Wibbeler et al., 2021). Pathophysiologically, all NCL types feature lysosomal dysfunction, neuroinflammation, and neuronal apoptosis; however, CLN2 is distinguished by specific TPP1 enzyme deficits leading to accelerated neurodegeneration. For instance, CLN2 commonly involves a splicing variant (c.509-1G>C) and a nonsense variant (c.622C>T, p. Arg208X), both of which severely reduce TPP1 activity. Pathogenesis centers on lysosomal dysfunction, disrupted vesicle trafficking, synaptic impairment, and neuroinflammation. Despite extensive research, the precise cascade from enzyme loss to neuronal death remains incompletely understood (Zhang et al., 2025).

Late-infantile neuronal ceroid lipofuscinosis (CLN2) is characterized by onset between ages 1 and 4 years, presenting with early language delay, seizures, ataxia, rapid cognitive and motor decline, and blindness. Most patients lose fundamental motor, speech, and visual functions and die between ages 7 and 15, though atypical variants with residual enzyme activity, late onset (up to 6 years), and slower progression are increasingly recognized. Clinically, EEG often reveals photosensitivity at low-frequency photic stimulation, as well as paroxysmal generalized discharges, supporting early suspicion (Kaminiów et al., 2022; Cotman et al., 2013; Palmer et al., 2013; Wibbeler et al., 2021; Gowda et al., 2021). In contrast, CLN1 (classic infantile NCL) presents even earlier, typically between 6 months and 2 years, with rapid progression, microcephaly, seizures, developmental regression, and earlier death (2–6 years). MRI often displays thalamic hyperintensities and callosal atrophy, while EEG rarely shows photoparoxysmal response. CLN3 (juvenile NCL) presents between ages 4 and 7, generally with early visual decline leading to blindness, followed later by cognitive deterioration, behavioral issues, seizures, parkinsonism, and cardiac conduction abnormalities. MRI shows both cerebral and cerebellar atrophy, but with a slower disease course than CLN2. Death typically occurs during adolescence or early adulthood (Kousi et al., 2012; Cotman et al., 2015; Zhang et al., 2025; Gowda et al., 2021; Mink et al., 2013).

Pathologically, shared features across NCL types include lyosomal accumulation, neuroinflammation, and neurodegeneration, though onset and progression vary depending on specific gene. Therapeutically, CLN2 is currently the only NCL subtype with an approved disease-modifying treatment. Unfortunately, similar options are currently unavailable for other NCL types, as no comparable specific treatments exist for CLN1, CLN3 and other forms. In their case, supportive care and seizure management remain essential (Kaminiów et al., 2022; Cotman et al., 2013; Wibbeler et al., 2021; Kohlschütter et al., 2019).

Although Batten disease most commonly affects children under the age of 10, the onset can range from birth to 30 years of age. Due to significant genetic heterogeneity and phenotypic variability, the diagnosis of NCL remains challenging. Here, we present the case of a patient with severe NCL type 2, emphasizing the diagnostic challenges encountered and exploring potential solutions to improve early detection and management.

## 2 Case presentation

The patient presented in this case report is a male child born in 2010 to healthy, unrelated young parents. He has a healthy older sister and a healthy younger half-sister. The family history was unremarkable. The boy was delivered via cesarean section and was the second child of his parents, although the third pregnancy. The first pregnancy ended in fetal demise in the first trimester, and the reason for the miscarriage was never diagnosed. The boy was born at 41 weeks of gestation, with an Apgar score of 10 at both 1 and 5 min, a birth weight of 4 kg, and a head circumference of 33 cm. His perinatal course was complicated by congenital pneumonia.

From birth, excessive tongue protrusion was observed. As a result, during infancy, he was evaluated by a speech therapist and a neurologist. No abnormalities were detected on clinical examination, and no further interventions were deemed necessary at the time. His motor development was age-appropriate: he sat independently at 6 months, crawled at 7 months, and walked independently by 10 months. However, speech development was delayed. He spoke his first words at 12 months, and by 18 months (until the onset of neurological symptoms) he used approximately six simple words.

Infancy was also marked by frequent respiratory tract infections, primarily obstructive bronchitis. Consequently, he came under the care of an allergologist at the age of 2 and was diagnosed with bronchial asthma. Since infancy, his parents reported hypersensitivity to external stimuli and suspected attention-deficit/hyperactivity disorder.

At 3.2 years of age (July 2013), he experienced his first seizures, which were focal in origin with secondary generalization. These were accompanied by unsteady gait, loss of consciousness, tonic body extension, and head deviation to the left, often resulting in falls. Oral automatisms (e.g., smacking), facial bruising, and vomiting were also noted. His first seizure lasted approximately 5 min and was followed by marked agitation and postictal sleep. A second seizure occurred during hospitalization for a respiratory tract infection. In addition to the infection, clinicians noted microcephaly, generalized hypotonia, and psychomotor hyperactivity.

**FIGURE 1:**
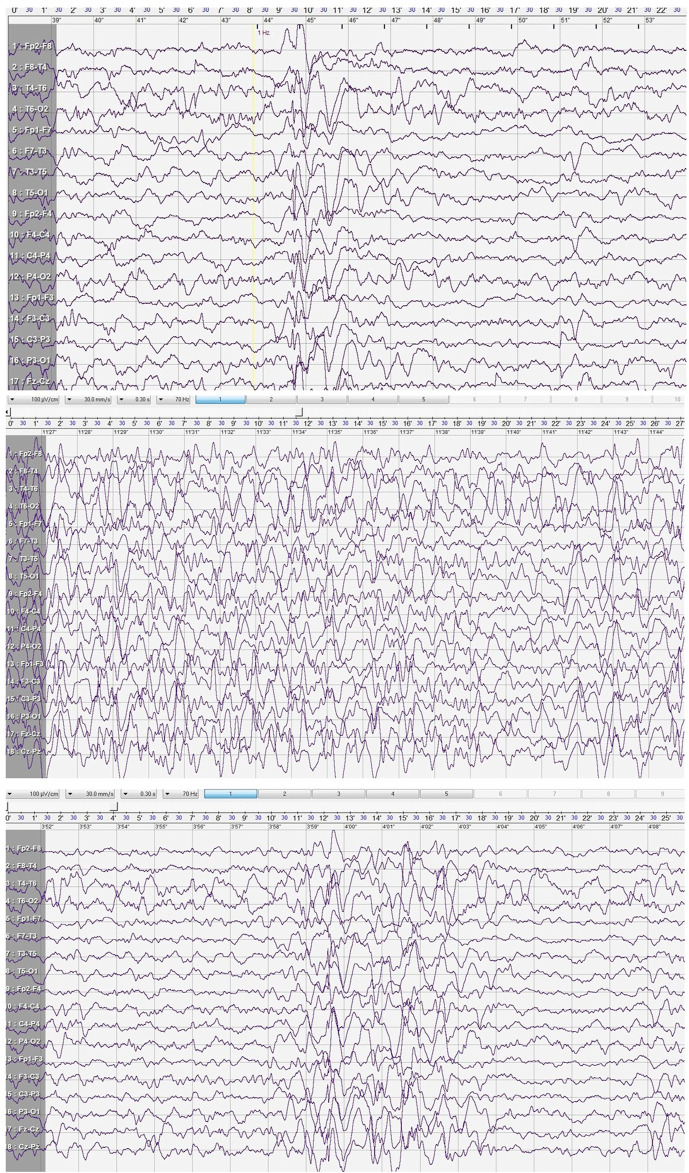
Electroencephalogram after 2^nd^ seizure.

A cranial CT scan revealed normal brain morphology, with only minor inflammatory changes observed in the paranasal sinuses. Following the second seizure, an EEG was performed during sleep. The study showed poorly developed sleep architecture and revealed paroxysmal discharges that lasted up to 4 s in the central and mid-temporal regions. These discharges consisted of high-voltage spikes, sharp waves, and sharp-and-slow wave complexes with amplitudes reaching up to 580 μV. The abnormalities were periodically generalized and were activated by low-frequency photic stimulation (Figure 1).

After the third seizure (August 2013), valproic acid was introduced to the patient’s treatment regimen. An MRI scan performed on 10 October 2013, revealed dilation of the occipital horn of the right lateral ventricle and persistent inflammatory changes in the maxillary sinuses. At the same time, genetic and metabolic diagnostics were initiated. Cytogenetic analysis showed a normal male karyotype, and microdeletions were excluded. Due to insufficient seizure control, levetiracetam was introduced while maintaining a therapeutic valproic acid level (93.69 μg/mL).

At 3.8 years of age (January 2014), neurological examination revealed subtle pyramidal paresis of the lower limbs and clumsy motor coordination. A 2-month period of seizure remission was also noted. However, by the age of 3.10 years (March 2014), seizure activity intensified again, with a predominance of prolonged tonic seizures and generalized tonic-clonic seizures. The addition of topiramate led to a brief improvement and reduction in seizure frequency, lasting only 4 weeks. During this period, upper limb tremors were also observed.

A psychological assessment conducted at the Psychological and Pedagogical Counselling Centre in May 2014 identified delayed psychomotor development, with cognitive function consistent with mild intellectual disability. At 4.1 years of age (June 2014), the child was referred to the Pediatric Neurology Department in Katowice to expand neurometabolic diagnostics. MRI at that time showed discrete widening of cerebellar grooves and the cisterna magna, but the brain appeared otherwise normal. EEG demonstrated generalized abnormalities, predominantly in the mediotemporal and posterotemporal-occipital regions.

Due to worsening balance issues, features of ataxia, and persistent seizures, suspicion of neuronal ceroid lipofuscinosis (NCL) arose at 4.5 years of age. MRI findings remained stable. A video EEG recorded generalized paroxysmal discharges lasting 1–3 s, consisting of high-voltage delta and theta waves, sharp waves, and sharp-slow wave complexes, which correlated clinically with generalized myoclonus, forward trunk tilting, and impaired consciousness. These were activated by photic stimulation. At that time, fundoscopic examination, as well as ENG and EMG, were normal.

A diagnosis of NCL was confirmed by markedly reduced activity of tripeptidyl peptidase 1 (TPP1) in leukocytes, measured at 0.61 U/mg protein/h (normal: 54 ± 18.2 U/mg protein/h). Consequently, epilepsy treatment was revised: topiramate and levetiracetam were discontinued, and lamotrigine was introduced.

At 4.9 years of age (February 2015), the diagnosis of NCL type 2 was genetically confirmed via direct sequencing of the TPP1 gene, which revealed a homozygous CGA→TGA mutation, resulting in a premature stop codon at position 208 of the protease polypeptide chain.

Following confirmation of the diagnosis, genetic counselling for future pregnancies and prenatal genetic testing was offered to the patient’s mother; however, she declined due to the father’s death and her decision not to have more children in the future. The patient’s sister underwent genetic testing and was not found to be a carrier of the mutation.

**FIGURE 2:**
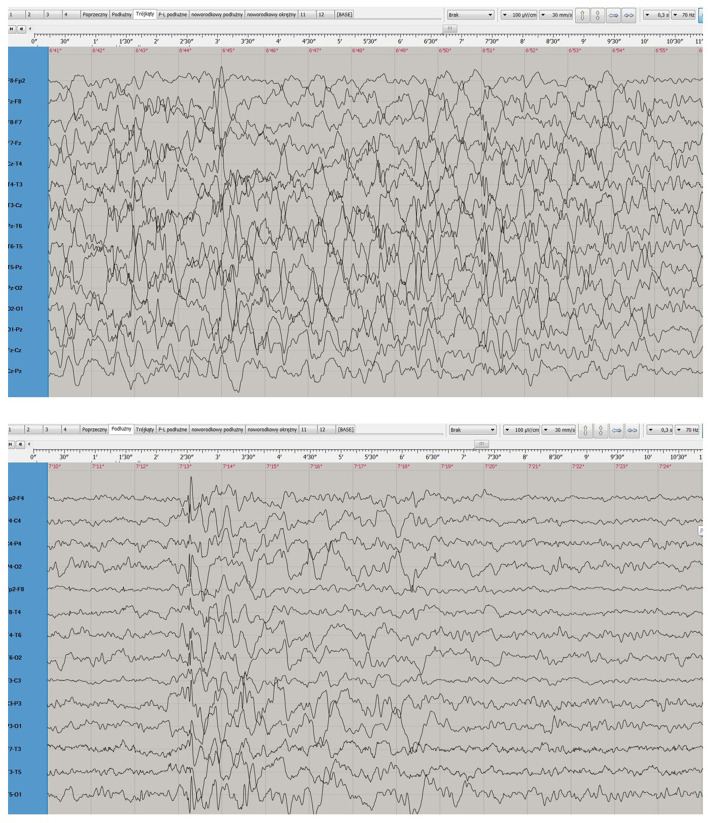
Electroencephalogram after the progression of the disease.

During a subsequent hospitalization in the Pediatric Neurology Department in Katowice (May-June 2015), triggered by an increase in seizure frequency despite multiple treatment modifications, MRI showed progressing cerebral atrophy with widening of the ventricular system and pericerebral spaces. An ophthalmologic examination revealed visual deterioration and optic nerve atrophy. EEG conducted during sleep, while the patient was receiving polytherapy (valproic acid, lamotrigine, levetiracetam, and clonazepam), showed poorly defined sleep patterns and diffuse slow activity dominated by 1–3 Hz waves (Figure 2).

In the following months, the child’s seizure frequency increased, with episodes of near-continuous myoclonus, prolonged tonic seizures accompanied by oxygen desaturation, and atonic seizures. These seizures were resistant to all subsequent modifications in treatment. Simultaneously, the patient’s motor and cognitive functions continued to deteriorate. At the age of 5 years and 11 months (March/April 2016), the child required admission to the intensive care unit (ICU), where a gastrostomy was performed. He was placed in a pharmacologically induced coma, which failed to produce clinical improvement.

The final antiepileptic regimen included valproic acid, ethosuximide, phenobarbital, topiramate, clonazepam, and haloperidol. The patient was eventually discharged for hospice care, initially inpatient and later continued at home. During his final hospitalization at the Pediatric Neurology Department, at the age of 7, EEG demonstrated low-voltage activity dominated by delta and theta slow waves, along with generalized discharges lasting 0.5–3 s. These discharges, with maximal amplitude up to 200 µV in the frontal regions, were unresponsive to photic stimulation.

**FIGURE 3:**
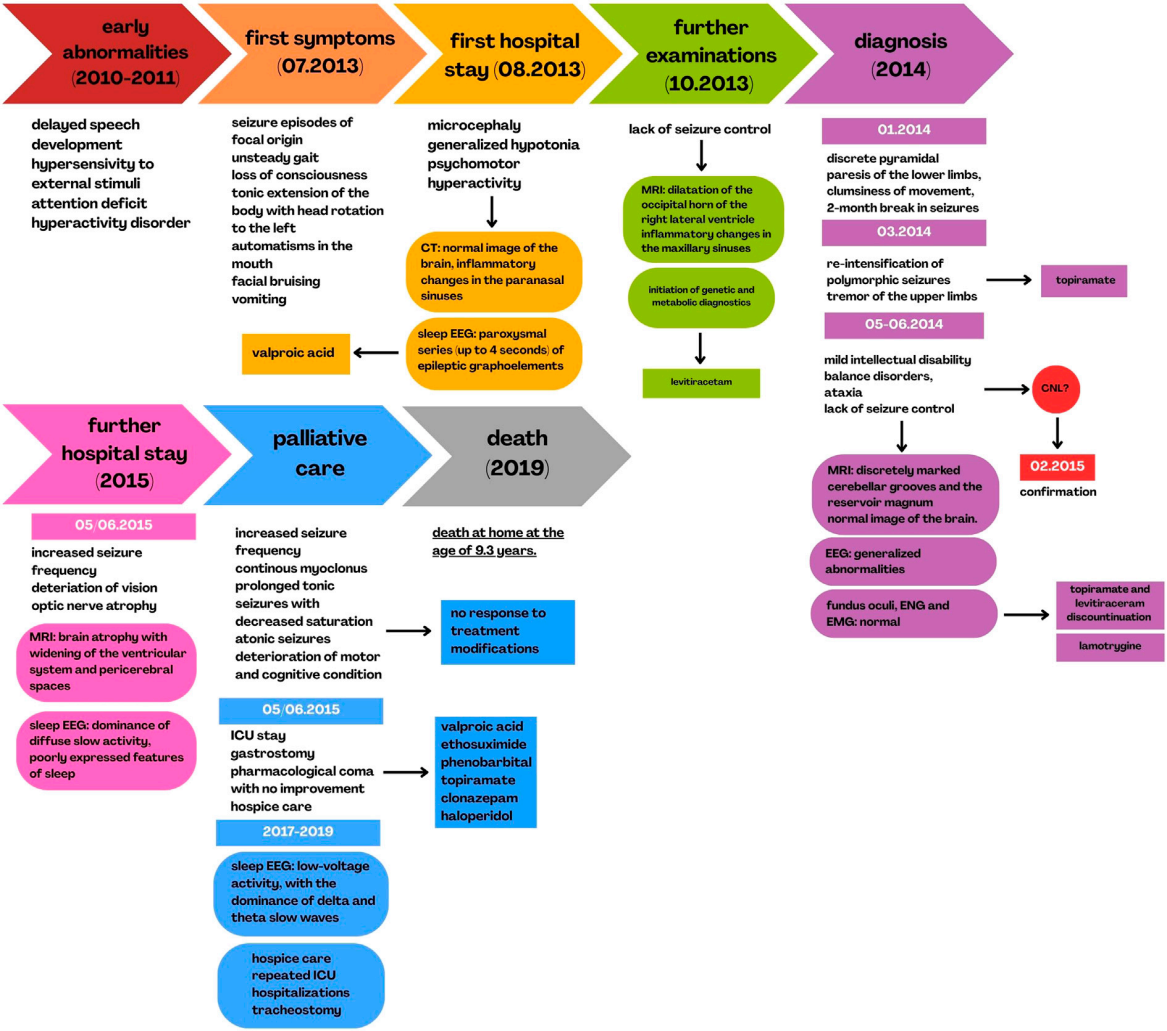
History of disease progression and diagnostics.

Following discharge, the child remained under home-based hospice care. He required repeated ICU admissions at his local hospital due to respiratory failure secondary to recurrent respiratory tract infections. At 8 years and 7 months of age, a tracheostomy was performed. The patient died at home at the age of 9 years and 3 months. The clinical and diagnostic course of the disease is summarized in Figure 3.

A generalized course of the patient’s symptoms, as well as the diagnostic process of the disease, including a description of imaging examinations performed during hospital stays, correlated with the timeline.

## 3 Discussion

Autosomal recessive (AR) conditions require both parents to be carriers of pathogenic variants in the same gene. While consanguinity increases the likelihood of such events due to shared ancestry, AR disorders can also occur in non-consanguineous couples. Studies estimate that every individual carries 1–2 pathogenic variants in AR disease genes, and up to 5–7 when considering broader genomic data, leading to a risk of approximately 1 in 200–300 births being affected by a recessive disorder even in unrelated couples (Biesecker, 2016). The likelihood increases with population-specific founder mutations or reduced gene pool diversity. For rare diseases like neuronal ceroid lipofuscinoses (NCL), this risk remains significant due to the extensive mutational heterogeneity and autosomal recessive inheritance patterns observed in nearly all NCL types (Kousi et al., 2012; Mole and Cotman, 2015). Therefore, carrier screening and genetic counseling are important preventive tools not only in consanguineous but also in non-consanguineous couples.

For NCL type 2, the only treatment able to stop the neurodegeneration process is cerliponase alfa (known under the name Brineura), a recombinant TPP1 enzyme. It is delivered via intracerebroventricular infusion to directly target neuronal lysosomes. In pivotal trials, treated patients showed marked reduction in motor-language decline compared to natural history controls (mean annual decline of 0.27 vs. 2.12 points). The therapy received FDA approval in 2017, with EMA approval following in 2017, restricted to patients aged ≥3 years. Ongoing studies are expanding to younger children and evaluating adjunctive early interventions (Schulz et al., 2013; Specchio et al., 2020). Cerliponase alpha was introduced to Europe in 2019 (Schulz et al., 2018). By this time, our patient had reached a late stage of the disease and died. Unfortunately, by the time of his diagnosis, he would also be excluded from the treatment outside Europe per the treatment criteria, as those disqualify candidates with severe neurodegeneration (akin to Schulz criteria in the clinical trails).

Apart from Brineura, symptomatic treatment is often administered. Consensus-based guidelines recommend gradual titration of anti-epileptic drugs (AEDs), like valproate, levetiracetam, lamotrigine, clonazepam, and topiramate, often progressing from monotherapy to polypharmacy due to refractory seizures. In severe cases, phenobarbital or midazolam may be considered during status epilepticus. Movement disorders in CLN2, including epileptic and non-epileptic myoclonus, dystonia, choreoathetosis, and spasticity, often emerge after seizure onset. Management involves a combination of AEDs and supportive therapies. Non-pharmacological support, such as physiotherapy, orthoses, weighted blankets, and positioning aids, may reduce symptoms and improve comfort. Interventions often also include management of sleep disturbances (Simonati and Williams, 2022; Radić Nišević et al., 2024; Löbbecke, 2019; Geraets et al., 2016).

Clinical trials using adeno-associated virus (AAV)-mediated CLN2 gene transfer (e.g., BMN 190) are underway, building on preclinical successes in TPP1-deficient animal models. The first human gene therapy trials for other NCLs (e.g., CLN3, CLN5) are also emerging. Additional experimental modalities, including substrate reduction therapy, small molecules, stem cells, and oligonucleotide-based treatments are being explored (Cotman et al., 2015).

The clinical course of CLN2 (Batten disease) is marked by rapid and unremediable neurodegeneration, thus highlighting the necessity of early diagnosis, ideally before symptoms emerge. Our patient, like many others, was faced with a delayed diagnosis due to vague initial symptoms and lack of clinical knowledge. Reasons for delayed diagnosis vary per region, but most commonly are linked with fragmented care pathways, clinician lack of knowledge about rare epileptic encephalopathies, and a lack of access to genetic and enzymatic testing (Gittus et al., 2023). Solutions need to tackle these by consolidating infrastructure, education, and policy frameworks. The protracted diagnostic journey in CLN2 and rare diseases often stems from low awareness among clinicians, limited access to specialized diagnostics, and fragmented care pathways. This is not the characteristics of NCL only; all rare disease patients typically undergo multiple consultations, averaging 6–14 years before diagnosis (Gittus et al., 2023; Adachi et al., 2023; Fietz et al., 2016; Bhatia et al., 2024).

For example, it was recently demonstrated that even presymptomatic treatment with cerliponase alfa could delay the onset of symptoms in children with NCL type 2, as well as that the implementation of swift gene panel testing for epilepsy in children who present with speech delay or seizure onset in early life vastly enhances diagnostic timeframe and treatment prospects. (Schaefers et al., 2021; Leal-Pardinas et al., 2022). Fortunately, some recent studies have focused on improving methods that might allow earlier testing for NCL2. The LINCE project developed a diagnostic algorithm using dried blood spots to detect NCL2, demonstrating high specificity and reliability. This approach has the potential for inclusion in neonatal screening programs (Rodrigues et al., 2022). Furthermore, research indicates that enzyme activity measurements in dried blood spots, plasma, and leukocytes can aid in diagnosing NCL2 with specific assays developed for newborn screening, which gives hope for this method to be used commonly in the future (Itagaki et al., 2018). As in some children, including our patient, clumsiness and delay in speech development might emerge before other, more alarming symptoms pediatricians should be aware that those symptoms might indicate a serious disorder, and refer those patients to a neurologist immediately (Lee et al., 2003). Overall, advancements in genetic and biochemical research are enhancing the understanding and diagnosis of NCL type 2 and paving the way for improved patient outcomes. As the current treatment methods can only stabilize motor and language function, but cannot reverse damage already done by the disease, early diagnosis is crucial for patients to maintain psychophysical wellness and live without repercussions of having Batten disease for as long as possible (Schulz et al., 2018; Rodrigues et al., 2022).

To further prove our point, recent reviews have emphasized the critical importance of early recognition and diagnosis in CLN2, highlighting that a clinical pattern involving developmental regression combined with emerging epilepsy, often subtle in the initial stages, should prompt immediate neurological referral and comprehensive diagnostic testing. Early vigilance is essential because timely diagnosis and intervention, including initiation of enzyme replacement therapy, can significantly alter disease progression and improve quality of life. This case exemplifies the necessity of heightened clinical suspicion and rapid diagnostic workup to enable early therapeutic opportunities in CLN2 patients, potentially delaying irreversible neurodegenerative decline (Amadori et al., 2020; Mazurkiewicz-Bełdzińska et al., 2021).

Delays in diagnosis not only jeopardize patient outcomes but also place significant moral and emotional strain on families, limiting care choices and preventing informed decision-making. Recognizing caregiver needs and acknowledging emotional costs should be integral to holistic care. CLN2 patients’ families usually endure weeks or months of uncertainty, emotional exhaustion, and social isolation during the diagnostic journey. Diagnostic inertia can be prevented by making caregivers active partners in the care process, providing early psychosocial care, and educating primary care professionals. Caregivers of CLN2 children report high stress, sustained vigilance, financial hardship, and social isolation (Gittus et al., 2023; Adachi et al., 2023; Ha et al., 2019; Great Ormond Street Hospital, 2019). As disease progresses, caregivers often become “expert advocates,” coordinating fragmented services, leading to exhaustion and burnout. Interventions such as caregiver training, psychological support, structured peer networks and shared coping are necessary to improve their quality of life. Not only caregiver’s quality of life is a value in itself; it is also strongly related to the quality of care they provide for their affected relative (Adachi et al., 2023; Ha et al., 2019; Dellve et al., 2006; Pelents et al., 2015; Steele and Davies, 2006).

Caregivers, particularly parents, play a pivotal role in the early detection of CLN2, often being the first to observe subtle developmental changes or emerging neurological symptoms. Validating caregivers’ concerns and incorporating their observations into clinical evaluation can facilitate earlier diagnosis. The emotional impact on families is profound, and supporting caregivers throughout the diagnostic and therapeutic process is ethically imperative (Mole et al., 2025; Schulz et al., 2020). Engaging families as active partners in care not only improves early recognition but also enhances adherence to treatment plans and optimizes overall management. Recognizing the caregiver’s perspective helps to bridge the gap between initial symptom onset and clinical intervention, reducing diagnostic delays and improving patient outcomes (Schulz et al., 2025; Williams et al., 2017).

As stated before, despite the fact NCL type 2 is an extremely rare disease, a quick and well-targeted diagnosis is crucial. Enzyme replacement therapy is currently available as the only therapeutic option, providing affected children with the opportunity to live a life of wellbeing. Early detection of this condition can provide the patient and their family with a chance to lead a normal life.

Ultimately, our case illustrates the disastrous outcome of delayed diagnosis in CLN2 and emphasizes the need for a more active, consolidated, and compassionate approach to treating rare pediatric neurodegenerative diseases.

## Data Availability

The raw data supporting the conclusions of this article will be made available by the authors, without undue reservation.
